# Extensive enactivism: why keep it all in?

**DOI:** 10.3389/fnhum.2014.00706

**Published:** 2014-09-25

**Authors:** Daniel D. Hutto, Michael D. Kirchhoff, Erik Myin

**Affiliations:** ^1^Department of Philosophy, School of Humanities and Social Inquiry, University of WollongongWollongong, NSW, Australia; ^2^Department of Philosophy, School of Humanities, University of HertfordshireHatfield, UK; ^3^Department of Philosophy, University of AntwerpAntwerpen, Belgium

**Keywords:** enactivism, functionalism, embodied cognition, mental representation, extended mind

## Abstract

Radical enactive and embodied approaches to cognitive science oppose the received view in the sciences of the mind in denying that cognition fundamentally involves contentful mental representation. This paper argues that the fate of representationalism in cognitive science matters significantly to how best to understand the extent of cognition. It seeks to establish that any move away from representationalism toward pure, empirical functionalism fails to provide a substantive “mark of the cognitive” and is bereft of other adequate means for individuating cognitive activity. It also argues that giving proper attention to the way the folk use their psychological concepts requires questioning the legitimacy of commonsense functionalism. In place of extended functionalism—empirical or commonsensical—we promote the fortunes of extensive enactivism, clarifying in which ways it is distinct from notions of extended mind and distributed cognition.

There are people of intelligence who can *learn* as many of

the facts of science as they like, but

… they lack the spirit of science.

For them it is enough to have discovered any hypothesis at all concerning any matter,

then they are at once on fire for it and believe the whole thing is accomplished.

To possess an opinion is to them the same thing as to become a fanatical adherent to it,

and henceforth to lay it to their heart as a conviction …

Insofar as genius of every kind maintains the fire of convictions and awakens distrust of the modesty

and circumspection of science, it is an enemy of truth,

no matter how much it may believe itself to be truth's suitor.

- Nietzsche, *Human all too Human* I: 635

## Getting beyond extended minds

After years of debate, philosophers, and cognitive scientists are still divided over the question of the extent of cognition. Does cognition happen entirely in the brain or is it, instead, a dynamic and interactive phenomenon that constitutively involves the environment, both spatially and temporally? Clark and Chalmers (1998) created recent interest in this discussion in their seminal paper “The Extended Mind,” famously defending the thesis that, at least in some cases of cognition, the world plays a constitutive role. Critics of the Extended Mind thesis agree that external, environmental interactions matter for enabling and shaping cognition in non-trivial ways, but they insist that exogenous influences make nothing but purely causal contributions (e.g., Adams and Aizawa, 2001, 2008, 2010; Fodor, 2009)—that, at best, non-neural factors play a merely supportive role in shaping and enabling cognition. The energetic production of publications arguing back and forth over the issue, wave after wave, is a sign that the extended mind debate has not been settled in favor of any of the contending parties.

In *Radicalizing Enactivism*, Hutto and Myin (2013) articulate and defend a radically enactive and embodied—thoroughly non-contentful—vision of basic cognition (REC for short)^1^. Enactivism is inspired by the insight that the embedded and embodied activity of living beings provides the right model for understanding minds. According to the radical version of enactivism defended in Hutto and Myin (2013), the vast sea of what humans do and experience is best understood by appealing to dynamically unfolding, situated embodied interactions and engagements with worldly offerings. Radical enactivism thus goes against the commonly held position that the best explanation of cognition always and everywhere requires positing contents that are acquired and transformed in order to create representations that then inform and guide what an organism does or experiences. Contents are understood as ways of representing the world that have conditions of satisfaction. As Crane observes: “To say that any state has content is just to say that it represents the world as being a certain way. It does have … a ‘correctness condition’—the condition under which it represents correctly” (Crane, 1992, p. 139).

Hutto and Myin (2013) defend the view that accepting REC has the advantage of enabling us to transform the terms of the extended mind dispute in the philosophy of mind. Specifically, its seventh chapter argues that endorsing REC provides both a spur and the sufficient means of moving beyond the “extended mind” debate. The book's diagnosis is that a fundamental obstacle to progress in the extended mind dispute is a continued commitment to the idea that cognition is necessarily content involving (CIC for short), something that has been accepted by those on both sides of it (Hutto and Myin, 2013, pp. 135–136). According to a REC analysis, if we let go of the CIC assumption our understanding of the extent of cognition is transformed, requiring a complete reconceiving of the standard extended mind debate. If we are correct, giving up on the idea that minds are essentially representational—always and everywhere content involving—is a game changer both for defenders of the extended mind theory and its internalist opponents. The reasoning is straightforward. Assume that basic cognition is not representational in a contentful sense. If so, then the clearest and cleanest grounds for internalism are undercut. For if basic cognition is not inherently contentful then defenders of internalism lose their most compelling reason for supposing that cognition—at least in its primary phylogenetic and developmental forms—is an “inner” business (where “inner” is understood in the sense of implying a cerebral location of the vehicles of content). Of course, many defenders of the extended mind theory also accept or presuppose that cognition is content involving. They disagree about the location and extent of the vehicles of such content. From a REC perspective the vehicle/content distinction does not apply at the level of basic minds: where there is no content there are no vehicles of content. Thus, if it turns out that basic forms of cognition are not contentful the extended mind debate should be transformed.

These observations about how the extended mind debate would be transformed if cognitive science adopted radical, non-representationalist theories of basic cognition seize on the fact that the standard, and strongest, move internalists can make to motivate their position is to appeal to a notion of narrow or intrinsic content (Adams and Aizawa, 2010). The appeal to mental content features crucial in internalist arguments because it is needed to provide a principled “mark of the cognitive”—one which backs up and gives definition to demarcation claims about what is constitutive of, as opposed to merely causally supportive of, cognition. This assumption is widespread and easy to find in the literature. The following quotations epitomize familiar sentiments about the representational mark of the cognitive and its perceived importance:

Admittedly, delimiting the scope of the “cognitive” is not an easy matter, but … it seems adequate to specify that cognitive states, structures, and capacities are mental entities with representational content (Khalidi, 2007, p. 93).

Without representation cognitive science is utterly bereft of tools for explaining natural intelligence. We would go further: without representation there is no cognitive (as distinct from behavioral, biologic, or just plain physical) science in the first place (O'Brien and Opie, 2009, p. 54).

It seems that anyone with a stake in debates about the extent of cognition and who abandons the representationalist “mark of the cognitive” must supply a tenable alternative; otherwise there is no clear cut way of distinguishing the cognitive from the non-cognitive.

Ultimately, we agree with O'Brien and Opie (2009). In the absence of an appeal to content there is no obvious alternative way to ground claims about what constitutes the cognitive in a scrupulous scientific manner. As we aim to show, one consequence of this is that without appeal to a notion of content to supply the mark of the cognitive—or an adequate replacement notion that can play that role—there is no principled way to advance the claim that cognition is neurally based. How else, other than by appeal to content, might the claim that cognitive processes are “contained in the brain” be supported? How else could it be established that, as a matter of fact, cognition is always and necessarily brainbound? Appeals to facts about brains or behavior on their own, lacking supplement by a substantive theory of cognition—we will argue—do not warrant that conclusion. We will return to this.

In all, from a REC perspective, to let go of the idea that basic cognition is necessarily contentful and representational in character is to remove a standardly assumed barrier to seeing at least this form of cognition as constitutively world-involving. Such a shift in perspective is tantamount to acknowledging that fundamental cases of perceiving and thinking are not necessarily grounded in, nor do they take the form of, representing aspects of the world or having contentful thoughts about those aspects. And to think this aligns perfectly with understanding cognitive processes as a form of wide reaching activity that is—at root—extensive and unbounded; thus extensive minds are not merely, occasionally and in special circumstances, extended.

## Insufficiency and irrelevance

Not everyone agrees that going radically enactive about cognition has these implications for the debate about the extent of cognition. In this section we focus on two charges that have been leveled, not against the truth of REC, but against its significance in this domain.

Wheeler (2014) claims that enactivism, even its non-representational variants, lacks the requisite theoretical resources to see off internalism about the machinery of cognition (or as he describes it the “whereabouts of our cognitive architecture”). He maintains that REC's claims about the extensive and constitutively world-involving relationality of basic cognitive acts are insufficient for rejecting internalism: by his lights, when it comes to putting internalism out of business REC “falls short of what is needed” (2014, p. 1)^2^.

He holds, by contrast, that extended functionalism—all on its own and without the aid of non-representationalism—suffices for rejecting internalism: extended functionalism, if he is right, does the trick whether it endorses representationalism or not. Consequently, he claims that extended functionalism can carry the day in the extended mind debate while remaining studiously neutral or agnostic about the question of the representational nature of cognition. In taking this line Wheeler denies the strong connection that RECers see as holding between representationalism, how we ought to understand the extent of mind and the rejection of internalism^3^.

Sutton (2014) also doubts that non-representationalism is relevant to debates over the extent of cognition. He holds that whether internal representations are assumed to exist or not is orthogonal to the internalism-externalism dispute—at least, if one's unit of analysis is cognitive activity understood as a public, distributed process. If Sutton is right, explanations of distributed cognitive activity (his examples are taken from the science of memory) can proceed without our having to get clear about, let alone settle questions about the existence (or otherwise) of mental representations. Although he doesn't always explicitly pitch his detailed discussions in terms of philosophical debates about mental representations, for him it remains an open question to what extent, for example, a “radically revised notion of the internal memory trace” commits theorists to mental representationalism (Sutton, 2014, p. 5). We need not be delayed by debates about the representational nature of mind in order to move memory research ahead, for example, since the real action in cognitive science does not depend on answering questions about whether minds represent or not. Hence Sutton's verdict is that debates about the existence or otherwise of mental representations are orthogonal to moving forward in (a) the internalism-externalism debate and (b) the explanatory project of understanding cognitive activity as a socio-culturally distributed process.

We disagree with both Wheeler (2014) and Sutton (2014). But before scrutinizing their views, let's consider, in order to set aside, a possible diagnosis of why non-representationalism may be thought to be irrelevant to the extended mind dispute. It might be thought that the issue of mental representationalism is entirely beside the point. Why think so? Here's a line of reasoning. Externalism and internalism take different forms depending on one's theory of mental representations. Different varieties of representational theory of mind assume that mental representations, whatever specific properties they happen to have, must—as a class—have *content* (of some kind) and *vehicles* (of some kind) if they are to qualify as representations at all. The qualification “of some kind” is important for it reminds us that it is sufficient only that the vehicle-content distinction is in play: it does not matter which particular form it takes. Against this backdrop it is possible to capture the main theoretical combinations in the internalism-externalism debate using a four-fold matrix. One can pair: (i) vehicle-internalism with content-internalism (Fodor, 1990); (ii) vehicle internalism with content-externalism (Fodor, 1994; Dretske, 1995); and (iii) vehicle-externalism with content-externalism (Wilson, 2004; Rowlands, 2010). It is also logically possible to pair (iv) vehicle-externalism with content-internalism (though we know of no one who adheres to this view).

What does this set of possible pairings show? One answer is that it reveals that questions about mental representations are orthogonal or irrelevant to what one thinks about the extent of cognition precisely because in adopting any one of the four combinations about representational vehicles and content won't settle the debate. In this light, progress in the extended mind debate can seem to require giving consideration to factors and features of cognition other than those concerning mental representations.

But it would be a mistake to conclude from this line of reasoning that the outcome of debates about whether cognition involves mental representations is irrelevant to how to best understand the extent of mind. Why? Firstly, if decisive arguments could be found to favor one of these four theories of mental representation—giving compelling reason to favor it over its rivals—this would bring discussions about the extent of mind to a close. So, far from being irrelevant, considerations about mental representations look as if they lie at the very heart of this dispute, and the fact that we cannot decide between the existing candidate views is in part what sustains debates about the extent of mind. Secondly, and relatedly, if representationalism is rejected *tout court*—if the issue of whether cognition is representational *at all* is moot—then the common ground for the extended mind debate, as characterized by this matrix, collapses under the very feet of all parties.

Consider an analogy. Several political parties disagree about the appropriateness of introducing new tax laws—each canvassing different combinatorial options. None of these parties question the need for taxation. Of course, merely recognizing that there is a need for taxation would not decide which combined tax law should be adopted. Other considerations would have to be brought to bear. But that hardly makes questions of taxation irrelevant or orthogonal. The choice between the various tax options on offer rests on the unquestioned assumption of a need for taxation. Foundationally, the imagined disputants agree on the need for taxation—hence taxation could not fail to be relevant to their political debate: it is the very basis upon which that debate is conducted. And, obviously, were taxation to be abolished the entire debate about different possible tax laws would be pointless. By the same token, it is difficult to see how one's stand on mental representation could be irrelevant or orthogonal to the extended mind debate and our best understanding of the extent of mind.

## From extended functionalism to extensive enactivism

Let's approach the question from a different angle: how might we understand the extent of mind if we adopt a non-representationalist extensive enactivism and what might motivate one to adopt such a position? First things first. In Hutto and Myin (2013), it was argued that understanding cognition as extensive and not extended is a consequence of adopting REC. Yet questions have been raised about the very idea of extensive minds that REC recommends. Wheeler (2014) wonders: “What does this [notion of extensiveness] mean?” (p. 1). By way of reply it is important to note that coining the label “extensive” was meant as a corrective—it was designed to highlight both what is, at once, right and wrong with the “extended mind” metaphor. How are the notions different? Wheeler captures the subtlety perfectly, when he asks: “is mind a phenomenon whose primary ontological manifestation is inner, but which sometimes spreads beyond the skin (extended functionalism as often understood), or a phenomenon that is widely constituted in its very essence (the extensive mind of radical enactivism)[?]” (p. 2).

As a first pass answer, we think Wheeler's rhetorical query captures the difference well enough. We will explicate the notion of extensiveness in greater detail in Section Extensiveness Explicated. For the moment we respond to those who think, despite admitting that the original “extended mind” terminology has its difficulties, there is no need to take up our neologism. Sutton (2014), for example, thinks following our lead on this score is unnecessary. As he sees it, other serviceable replacements are, and have long been, readily available in the literature:

It is true that the word “extended” can easily be misread as assuming a more basic inner cognitive system which only spreads later in development. For this reason, the well-established pre-existing label “distributed cognition” should be preferred (Hutchins, 2014): there is no need for the awkward enactivist coinage “extensive mind” (Sutton, 2014, p. 14).

We agree with Sutton that the label “extended mind” has problematic connotations. If the only choice is between “extended mind” and “distributed cognition” then we prefer the latter. But the notion of extensive minds not only captures what is right in the idea that minds are already world-involving in their basic forms, it also sets its face against internalism in a way that those who endorse the distributed cognition framework do not. Distributed cognition—as Hutchins defines, and Sutton endorses, it—denotes a pragmatic stance through which hypotheses about the extent of mind can be formed and tested. Thus, “to take the distributive perspective is *not to make any claim about the nature of the world*. Rather, it is to choose *a way of looking at the world*, one that selects scales of investigations such that wholes are seen as emergent from interactions among their parts” (Hutchins, 2014, p. 36, emphases added).

Understood as a pragmatic stance, distributed cognition may well be the right explanatory attitude to adopt. But what does adoption of this stance tell us about the extent of mind? Taking it up requires making and defending claims about the extent of cognition in specific cases in the light of empirical findings—for only then do the metaphysical questions have real grip. What does this rule in or out about the extent of minds in general? Answer: the extent of mind is shifty. In some cases and on some scales cognition is wide-ranging, on others it may be wholly internally constituted.

A central claim of the distributed cognition framework is that the proper unit of analysis for cognition should not be set a priori, but should be responsive to the nature of the phenomena under study. For some sorts of phenomena the skin or skull of an individual is exactly the correct boundary. For some phenomena, the whole person is just too big and including the whole organism would involve too many interactions. For other phenomena, setting the boundary of the unit of analysis at the skin will cut lines of interaction in ways that leave key aspects of the phenomena unexplained or unexplainable (Hutchins, 2010, p. 426).

Extensive enactivism is not, by contrast, compatible with internalism. We believe that theoretical considerations about the nature of information and the problems they raise for naturalizing content tip the balance in favor of non-representational theories of basic cognition. This, in turn, gives us reason to reject internalism, and—indeed—to move beyond the extended mind debate as traditionally formulated. By implication, it gives us reason to think that answers to the question of the extent of mind will not be shifty in the way fans of distributed cognition propose.

Is this to favor metaphysical pronouncements and posturing over empirically informed theorizing about cognition? No. The relation between philosophical and scientific contributions is complex. Following the lead of neo-Quineans, when it comes to understanding the nature and extent of cognition we think the philosophical job is two-fold:

The metaphysican has work to do, first, in helping to determine what our best theories are (weighing up the theoretic virtues of competing theories), and second, in determining what … those theories commit us to … The work is neither empirical nor conceptual (indeed those two cannot be separated …) (Thomasson, 2014, p. 107).

Philosophical considerations about the features of the best global theory of cognition, we think, tell against the idea that basic minds are contentful and this is what pushes us to adopt the notion of extensive minds. But to explicate the notion of extensiveness more thoroughly and motivate it properly we need to go beyond the first pass characterization offered above. For some will still find that characterization of extensiveness imperspicuous. There is a need, as Wheeler (2014) identifies, to say more about “precisely how are we to explicate the property of extensiveness, and thus what it means for mind to be ‘widely constituted in its very essence’?” (p. 2).

Paving the way for a more developed answer to Wheeler's question, let's begin by considering problems that beset various kinds of non-representationalist extended functionalism. Roughly rendered: non-representational functionalism, extended or otherwise, is a pure form of functionalism. Pure functionalism does not entail any kind of representationalism: pure functionalism is a weaker thesis than representational functionalism.

There are two ways of formulating a pure functionalism. One way is to adopt some kind of functionalism based wholly on empirical science. The second way seeks to establish functionalism's legitimacy by appeal to ordinary talk and thought about the mental. In what follows we will argue that any pure empirical functionalism, extended or not, that abandons representationalism without supplementing that loss with an alternative substantive theory of cognition, is inadequate as a means of demarcating the extent of the cognitive. By contrast common sense functionalism appears to have better prospects in this regard. However, if such a functionalism takes seriously (as it must) everyday thought and talk about cognition, then there are reasons for thinking of cognition as wide, world-involving phenomena yet, at the same time, a proper investigation of our everyday talk of the mental provides no justification for adopting a functionalist framework for understanding cognition in the first place.

In reviewing the fate of non-representational, pure extended functionalism in both its scientific and commonsense formulations we will ultimately be in a position to explicate the notion of extensiveness such that it will become clear why asking questions about boundaries of the cognitive is always a matter of asking questions that take stock of the world-relatedness of cognition.

## Extended functionalism via empirical functionalism

What case can be made for the extended mind by appeal to empirical, non-representational functionalism? Wheeler (2010) holds that even though pure functionalism does not suffice, on its own, to establish the truth of the extended mind hypothesis it nevertheless promotes the idea of multiple realizability. And the idea of multiple realizability, he holds, is pivotally important for making a case for the possibility of extended minds. Considerations about multiple realizablity, he holds, allow that it could turn out that “the borders of the cognitive system … fall somewhere other than the sensorimotor interface of the organic body. And that opens the door to a cognitive system whose boundaries are located partly outside the skin” (Wheeler, 2010, p. 249).

As a statement of what extended functionalism is and its conditions of possibility this is all well and good. Wheeler's analysis sets the stage for the extended mind debate, but without a substantive theory of cognition—one that provides an alternative non-contentful mark of the cognitive—empirically based functionalism only allows for the logical possibility that minds might extend—it does nothing more to promote the fortunes of extended functionalism.

In what follows we will show that without the backing of representationalism pure functionalism encounters intractable problems of definition and demarcation—it looses its principled basis for determining what cognitive “inputs” and “outputs” are, and thus for saying where cognitive processes might begin and end. Without a substantive theory of cognition to provide requisite backing it is not possible to determine the significance that various facts about brains, behavior or computation have for our thinking about the extent of cognition. Abandoning the idea that cognition is representational—content involving—leads step-by-step to an unthreading of empirical functionalism and ultimately brings it face-to-face with old and familiar charges of its triviality.

There is a familiar story about computers according to which computational operations are performed on various bits of information stored as unique strings of code. These bits of information are conventionally identified by a unique assignment of 0's and 1's and at the hardware level these “codes” are said to be physically realized in the computer's on-off states. This ensures that a computer's operations can be carried out purely mechanically. It also explains the great versatility of computers, which derives from the fact that these various sets of 0's and 1's can be given different semantic interpretations. In one computer programme what is found at a given register might be treated as a word, in another it might be treated as a number, or in yet another, as some geometric figure. That's the upside. The downside is that “If computation is defined in terms of the assignment of syntax then everything would be a digital computer, because any object whatever could have syntactical ascriptions made to it. You could describe anything in terms of 0's and 1's” (Searle, 1990, p. 26). Call this the problem of computational individuation.

A way around this problem is to make good on a theory of computation of the sort offered by Piccinini (2008)—one that understands computation in purely non-representational, functional terms. This requires giving a theory or explanation that is “sufficient to individuate computational states without appealing to either semantic or syntactic properties” (Piccinini, 2008, p. 209).

We agree that a theory of this kind is what is needed. But even if such a theory provides a basis for a pure functionalist account of computation there is a need to forge a further link so as to connect that purely functional account of computation to cognition. In assessing the prospects of discovering that link it is crucial to realize just how wide and unbridgeable the gap in question really is if one adopts a non-representational notion of computation.

We can get a grip on the magnitude of the problem by seeing how different and distant a purely functional theory of computational individuation is from either a representational or syntactic theory of mind. Consider that the received view takes it for granted that, “computational states are individuated at least in part by their semantic properties” (Piccinini, 2008, p. 205). Or, as Fodor more pithily puts it: “there is no computation without representation” (Fodor, 1981, p. 180). Or, again, as O'Brien and Opie (2009) maintain “computation is governed by the contents of the representations” (2009, p. 53).

When it comes to individuating mental states orthodox cognitive science has lived by the hope that the threat of triviality generated by the problem of computational individuation could be avoided by a different route than that proposed by Piccinini (2008). The problem might also be dealt with if the hypothesized symbol-strings had *bona fide* representational properties. If that were so classical cognitive scientists would have a toehold for claiming that a theory of cognition that postulates symbols, with both semantic and syntactical properties, might be true of organic brains.

If a unified computational and representational theory of mind could be developed to provide a justification for the realistic assignment of contents it could thereby individuate syntactic structures. This proposed link is clearest in the language of thought hypothesis that invites us to imagine, put crudely, “that human mental sentences are written on little cerebral CRTs” (Stich, 1983, p. 53). Yet what if we abandon the idea that the Mentalese sentences that are imagined to fill our heads are contentful? Can we just drop the idea that there is a relation between representational semantics and computational syntax by rejecting the former while hanging on to the latter?

In order to avoid familiar worries about the casual impotency of content, Stich (1983) proposed doing just that. He advanced a syntactical theory of mind according to which no such relation existed; that is to say, he proposed a retreat to a pure, non-representational functionalism. But to abandon the idea that mental states have semantic properties raises questions about the very notion of syntax. What does a proponent of this view imagine is left behind? Syntax and semantics, as classically understood, are internally related: thus, it is simply not conceivable that there can be talk of syntax in the absence of semantics. If so, it is not clear how we might individuate mental states, carving up the mind, in purely syntactical terms without appeal to semantic properties. Thinking that a separation between syntax and semantics is possible is a “common but serious mistake” (Piccinini, 2008, p. 208)^4^. It will only appear unproblematic that we can talk of syntactic properties independently of semantic properties if we imagine that syntax keeps its shape, somehow, as a kind of shadow of semantics. Yet once semantics goes, its shadow—syntax—goes too.

We are thrown back on the old problem that pure functionalism always faces: how are we to define and individuate mental states without appeal to representational properties? Certainly, we cannot do this by appealing to bottom-up facts of neuroscience in the absence of a substantive theory of cognition. Even Adams and Aizawa (2010) acknowledge that:

We have no way to identify particular tokens of brain states qua syntactic state items in order to affix contents to them. Given the state of current science, we only identify a person's brain states via inferences to the content of those states (p. 72).

Our analysis takes this observation a step further: we cannot identify a person's brain states qua syntactic states except by appealing to semantic properties. Once this is acknowledged it looks as if a retreat to pure functionalism leaves us bereft of the resources for individuating the cognitive. As the infanticidal father of functionalism writes of his once brainchild:

One looks for something definable in non-intentional terms, something isolable by scientific procedures, something one can build a model of … And this—the “mental process”—is just what does not exist (Putnam, 1988, p. 74).

Is this correct? Let's address this question by examining some recent proposals in the extended mind debate. Adams and Aizawa (2010) advance an internalist proposal about how to individuate the cognitive by appeal to two necessary conditions: (i) the intrinsic content condition and (ii) the causal processing condition. Accordingly “cognition is constituted by certain sorts of causal processes that involve non-derived content” (p. 68, see also Adams and Aizawa, 2001, pp. 52–53). Of course (i) has to go if we give up on representationalism. What's left? Could appeal to (ii) on its own provide secure basis for thinking that cognition is a constitutively heady affair without an appeal to content? Without CIC in play, are Adams and Aizawa right to think that the “weight of empirical evidence” favors cognitive intracranialism (p. 74)?

It is hard to see how this could possibly be so. For given how Adams and Aizawa set things up once we lose the first condition we are left only with the unilluminating thought that certain sorts of causal processes constitute cognition. That would be a kind of internalist pure functionalism. Perhaps this internalist functionalist proposal might be defended by appeal to the idea that we can demarcate which causal processes are the ones that matter by appeal to information processing differences. Yet, again, things get tricky if we give up on the idea that informational content is processed. Talk of information processing becomes less than perspicuous if no content is literally processed—once that favorite metaphor of cognitive science gives way (Hutto and Myin, 2013, ch. 4). Indeed, if information is understood in purely covariance terms then an organism's sensitivity and responsiveness to information will be a world-involving activity, as extensive enactivism would have it. What would warrant dividing up such a “process” into smaller cognitive and non-cognitive parts? How, without an appeal to content, could this lend any support for functionalist internalism? Adams and Aizawa (2010) recognize this problem themselves holding that unless an appeal to content is in play the idea “that information processing constitutes the mark of the cognitive … is implausible” (p. 76).

But perhaps it might be thought that all is not lost for internalist functionalism. What if there was a special way that brains respond to information intracranially—a way that, say, marks out such neural activity as being importantly different, setting it apart from the way brains behave when they are engaged in activities involving, e.g., extra-cranial brain-tool combinations? Rupert (2009) has developed arguments along these lines to support the claim that human cognition is located “inside the organism either entirely or in the main” (p. 45). To reach this conclusion he invokes the systems-based principle—a principle that makes no reference to representational content and thus suits pure functionalism. Accordingly:

[A] state is a cognitive state if and only if it consists in, or is realized by, the activation of one or more mechanisms that are elements of the integrated set [of members] which contribute causally and distinctively to the production of cognitive phenomena (2009, p. 42).

How does this help? There is a continuing, well known debate about the exact nature of mechanisms (Craver, 2007; Bechtel, 2008). Let us imagine the best case for Rupert and suppose that all goes well with recent work on mechanisms and that it yields a precise means of individuating them from the rest of nature. Let us also imagine that we can determine precisely when, where and which mechanisms are implicated in cognitive activity. Assume further that we are reliably able to discern which are the more, or even the most, integrated mechanistic parts of any given cognitive activity. Even in this perfect scenario it is not clear what would license treating the non-mechanistic or less integrated parts of the activity as non-cognitive. That verdict would be driven by appeal to the stipulated systems-based principle. But lacking a substantive theory of cognition—as opposed to a theory of mechanism—what independently justifies that conclusion? For this sort of proposal to succeed the connection between the mechanistic and the cognitive needs be clearly established^5^.

We can see the problem clearly by considering the fact that no one in the debates about the extent of cognition denies that cognitive activity involves mechanisms. What is questioned is why anyone ought to suppose that the cognitive is limited or restricted to certain, say, mechanistic or computational parts of such activity? Consider the analogy between perceiving and driving. Like driving, perceiving can be understood as a situated, environment involving activity—and nothing short of that. To be sure, driving depends, in part, on the activity and interactions between a set of integrated mechanisms. Moreover mechanisms are surely found within the car, but it does not follow from that fact that driving happens within the car. Nor is anything gained for mechanists by noting the fact that “one can manipulate the car's behavior by manipulating its engine” (Noë, 2004, p. 211). For it is, of course, equally true that one can intervene on the driving activity by “wiggling” the (presumably) non-mechanistic environmental features, such as the condition of the roads.

Without some further grounds for conferring cognitive status on integrated mechanisms, other than stipulation, we face essentially the same problem again: nothing justifies the assumption that mechanisms *per se* pick out the cognitive^6^.

The root issue is that even if mechanisms feature in cognitive activity this fact does not, by itself, tell us what significance such mechanistic activity has for demarcating the cognitive from the non-cognitive. To see this, note that what is intended to justify the systems-based principle is *not* a substantive theory of cognition; rather, it is the alleged empirical fact that it is by presupposing that mechanisms are where the *real* cognitive action resides is what best “accounts for the successful practice in cognitive psychology” (Rupert, 2009, p. 43). In fact, this defense of the systems-based approach rests on a number of disputable empirical claims. The most important are that: (1) orthodox cognitive psychology has been successful; (2) orthodox cognitive psychology individuates cognitive activity in terms of mechanisms; (3) that interesting laws of cognitive psychology are tied to finding mechanisms; and (4) the latter facts (2) and (3) conspire to best explain (1)^7^.

Let us suppose, for the sake of argument, that (1)–(4), as stated above, are all true. That supposition poses no threat to thinking that cognitive activity is extensive. For it may *also* be true that cognitive psychology is or will be successful because it individuates cognitive activity non-mechanistically and that it will discover other interesting laws that are not tied solely to mechanisms. There is no logical reason to exclude these possibilities in advance. Put otherwise, without a bona fide theory of cognition, there is no principled reason to suppose that the successes of cognitive psychology depend on mechanisms, and mechanisms alone. To think otherwise would be in the thrall of a fallacious induction. To illustrate: suppose that all results established in cognitive psychology to date have individuated cognitive activity in terms of mechanisms. Nevertheless, it would be a non-sequitur to assume that a more expansive, non-mechanistic mode of individuation would not yield significant and non-trivial results.

Dale et al. (2009) make a related point. Cognitive processes, they assert, are too complex and heterogeneous to suppose that one and only one framework can account for everything. Indeed, there are positive reasons to think that there is a need to go beyond the mechanistic and computational, narrowly conceived, and press for a radically enactive or embodied cognitive science. Chemero (2009) provides a clear and compelling case of the need to do so in his argument against neural reductionism that is “based on the details of experimental practice” (p. 170). He cites the unhappy situation in the study of exploratory behavior in animals in which cognitive psychologists systematically under-described features of object exploration tasks such that their findings, while not useless, were—to use Chemero's words—potentially confounded. The root problem is that—based on a survey of 116 papers—the majority of work in this area inherited protocols that did not detail the nature of the objects used, and the way they could be interacted with by the animals, in their experimental designs to an appropriate degree^8^.

This is important because, as it turns out, the exploratory activity of the species under investigation is greatly influenced by the possibilities for action that those objects afford them. For example, it matters very much to how an animal explores an object whether it has properties that allow the animal to climb it or not (as was explicitly demonstrated in experiments reported in Chemero and Heyser, 2009). In the words of Chemero (2009) “not all objects are created equal: their affordances really do affect the way animals explore them” (p. 175). By thus ignoring crucial differences the bulk of work in this area failed to “give enough information about the objects used, and so failed to meet one of the primary goals of scientific research: these studies are *not replicable*” (Chemero, 2009, p. 173, emphasis original). This is alarming given that molecular neuroscientists, behavioral geneticists and psychopharmacologists have relied on these “potentially confounded studies.” This is a clear, yet probably not isolated, case in which the need to go beyond the boundaries of traditional, orthodox cognitive psychology is evident (for a recent example that shows how attention to interactive properties of objects pays off empirically, see Morlino et al., 2014).

Proponents of representational accounts of internalism could respond to this problem, at least in principle. For if representations with various contents—contents that represent or stand in for the various affordance-like properties of different objects—feature in cognitive mechanisms then it might be possible to explain how the objects of environmental properties matter without going wide. But, importantly for our discussion here, if one rejects representationalism such a move is unavailable even in principle. For the version of pure extended functionalism currently under consideration the standard internalist line of reply is closed off because the latter line of reply assumes the truth of representationalism. Once representationalism is off the table, we have no choice but to accept that specific properties of objects and how organisms relate and respond to them will need to feature in, at least some of, our best scientific explanations of cognitive activity.

Importantly, extended functionalism plays no part in the above argument for blocking and undermining internalist pure functionalism. The last stage of our argument has two main steps. First, it notes that once one goes non-representational there is no clear scientific rationale for, and no clear theoretical means of, thinking of cognitive activity as something smaller or shorter than world-involving extensive relational activity. Second, it provides positive scientific reasons for going wide. Tellingly, the parade case we highlighted from Chemero (2009) works precisely because it rests on a substantial theory from ecological psychology—one that makes appeal to the notion of affordances. It is not a mere negative inversion of internalist functionalism, such that it assumes extended pure functionalism follows if internalist pure functionalism fails. The positive, substantial motivation for going wide in an extensive and not just an extended way does not rest on functionalist considerations. This should hardly come as a surprise for as Putnam explained long ago, when first introducing empirical functionalism, that it was only ever a framework for theorizing and advancing empirical hypotheses; in itself empirical functionalism is “the putting-forward, not of detailed scientifically ‘finished’ hypotheses, but of schemata for hypotheses” (Putnam, 1967/1992, p. 54). Yet—and here's the rub—if extended functionalism does not, by itself, motivate or play any part in convincing us to go wide—if it is only a hollow theoretical frame—we have both the option of dropping it and no obvious reason to retain it. In this light clinging to functionalism in the debates about the extended mind looks like nothing more than intellectual inertia.

## Extended functionalism via commonsense functionalism

So far so good. But, assuming the above arguments go through, the obvious move for the extended functionalist to make is to steer clear of empirical pure functionalism. Not only is this an obvious response—the extended functionalists who follow Clark (2008, 2011)—have prepared this answer in advance: for their extended functionalism is explicitly advanced under the auspices of commonsense functionalism. In a retrospective on his seminal paper with Chalmers, Clark makes clear his official view on the kind of extended functionalism he endorses “is better viewed as a simple argumentative extension of at least a subset … [which is non-committal about conscious states] … of what Braddon-Mitchell and Jackson (2007) describe, and endorse, as ‘commonsense functionalism’ concerning mental states” (Clark, 2008, p. 88). Or again, “It is the coarse or common-sense functional role that, on this model (unlike that of empirical functionalism), displays what is essential to the mental state in question” (Clark, 2008, p. 88).

However, extended functionalism is not (nor was it originally thought to be) an immediate or obvious consequence of commonsense functionalism. As formulated and advanced under the so-called Canberra Plan that embeds the commonsense functionalism of Lewis (1972, 1995), Jackson, (1998) and Jackson et al. (2009) and sought to reveal, through conceptual investigations, what the folk “find obvious” about the mental—by attending to and perspicuously representing what lies behind their thought and talk about, *inter alia*, the mental; viz., to reveal the content of our shared implicit folk theory of the mind. So conceived commonsense functionalism only works—it only has legitimacy—if it descriptively captures and states *only* what the folk find obvious about the mental and nothing more. The project depends on accurately characterizing our folk commitments. With this in mind Lewis instructed commonsense functionalists to:

Collect all the platitudes … regarding the causal relations of mental states, sensory stimuli, and motor responses. … Add also all the platitudes to the effect that one mental state falls under another … Perhaps there are platitudes of other forms as well. Include only the platitudes which are common knowledge amongst us: everyone knows them, everyone knows that everyone else knows them, and so on Lewis (1972, p. 256).

Given this backdrop it is not obvious that unadulterated commonsense functionalism ought to embrace extended functionalism. Consider that opinions are divided about how to respond to Clark and Chalmers' endlessly repeated case of Otto and his notebook. This shows that if we were only to look at the intuitions of the folk, as revealed by how they respond to possible cases, there is no obvious reason to favor extended functionalism over internalist functionalism. In discussing folk reactions to these kinds of thought experiment, Chalmers—in his foreword to Clark's *Supersizing the Mind*—admits that perhaps the opponents of the extended mind thesis “would have commonsense psychology on his or her side. If so, then perhaps this is one point where the ‘commonsense functionalism’ that Clark favors in this book, individuating mental states by the roles that commonsense psychology assigns to them, counts against the extended mind thesis” (Clark, 2008, p. xii)^9^.

In the light of this, Chalmers recommends the following strategy: “At this point, I think the proponent of the extended mind should not be afraid of a little revisionism. Even if commonsense psychology marks a distinction here, the question still arises of whether this is an important distinction that ought to be marked in this way” (xii). The obvious problem with this suggestion is that to steer clear of commonsense is to move away from the very project that mandates and motivates commonsense functionalism as described above. In its original formulation the only reason for accepting commonsense functionalism is that it is revelatory of what the folk think about the mental. Without that backing—in pressing for open revisions of what the folk think—extended functionalism would be working without a net. If extended functionalists cannot fall back on an appeal to science and empirical findings (see previous section), it is utterly unclear what could possibly warrant or legitimately constrain the necessary revisions.

Clark (2011) recognizes the insufficiency of appealing to purely scientific considerations that are untainted by commonsense to secure extended functionalism. He comments on Wheeler's (2011) empirical functionalist proposal for doing just that, saying: “This is a splendid idea, *but one that (it seems to me) is almost certainly doomed to failure*. The reason it is doomed to failure is that the shape of any such scientific theory of legitimate vehicles will surely be determined, in large part, by what we take as central examples of real-world realizers of cognitive processes in the first place.” (p. 452; emphasis added)

In this light, Clark (2011) maintains that individuating mental states—“finding the mind”—by appeal to coarse-grained behavior patterns using our folk psychological concepts is inevitable. He makes this commitment to commonsense indelibly clear, when he writes:

It is now over a decade since this question started being debated within philosophy and cognitive science. My current view (arising from the ongoing debates with critics such as Adams and Aizawa, Rupert, and influenced also by the rich and compelling recent treatment in Sprevak (2009) is that this debate, though scientifically important, and able to be scientifically informed, looks *increasingly unlikely to admit of straightforward scientific resolution* (p. 454; emphasis added).

In the final analysis, just like their empirical cousins, extended commonsense functionalists need more than the observation that commonsense tolerates the possibility that cognition might extend. To motivate extended functionalism via commonsense functionalism it must be established that careful attention to what the folk really think about the mental gives us some substantive, positive reason to believe in extended functionalism.

Where might we look for this? Arguably by examining more closely what the folk say and do when competently deploying their psychological concepts. However, it turns out that the best attempts to describe folk thought and talk about the psychological presents a serious challenge to functionalism. If we are properly attentive to the way the folk use their psychological concepts we will be driven to abandon functionalism, not to continue to work within its template. This is because functionalism it itself, arguably, a presumptuous imposition on commonsense (Ratcliffe, 2007; Hutto, 2011).

The truth of functionalism is, in fact, far from obvious. It is not at all clear that the functionalist framework is a natural part of commonsense psychology rather than something imposed on it. Moreover, attempts to defend functionalism by appeal to common, folk intuitions has raised important questions about the legitimacy of appealing to intuitions to secure important philosophical results in general (Fischer, 2011). Investigating and detailing how the folk think and talk about the psychological “is not an easy task … [and] the fruits of such an enquiry need to be distinguished from superficial and possibly widespread intuitions” (Ratcliffe, 2009, p. 380). A proper investigation into the folk use of concepts would take “considerable philosophical work to formulate, rather than a casual, uncontroversial statement of what the ‘folk’ think” (Ratcliffe, 2009, p. 382)^10^.

## Extensiveness explicated

Thankfully a body of detailed work examining the way our folk concepts of mind function in everyday contexts already exists. Wittgenstein's reminders about the role our concepts of perception play in our lives are a prime example. His philosophical investigations remind us of the circumstances in which we competently speak of “what is seen” and the many and various ways we have of responding to what is seen. These include diverse acts of description—such as creating representations of what is seen—where such representations are to be understood as acquired and sometimes quite sophisticated ways of acting and responding to what is seen. Distinguishing among these requires attending to “fine shades of behavior.” For this reason the concept of seeing is “internally linked with doing or being able to do, something, rather than with having something that we each of us know only from our own case” (McGinn, 1997, p. 203). McGinn provides a detailed, and mostly illuminating, analysis that everywhere stresses the indissoluble links that Wittgenstein reveals to hold between what it is to see and our ways of responding to what is seen (McGinn, 1997, pp. 195–204).

What we discover when examining the way our everyday psychological notions function in ordinary contexts is that “inner processes stand in need of outward criteria” (Wittgenstein, 1958, p. 50). This appeal to the importance of outer criteria is meant to remind us that the links between the inner and the outer are tight and intimate. Moreover the kind of behavior and activity that serves as a criterion for the psychological is not the mechanical or thoughtless variety; it is—as enactivists would have it—always and everywhere already enlivened, expressive and mindful. To adopt this view is to move beyond familiar, textbook models of the inner–outer relationship.

The very idea that psychological concepts pick out and describe phenomena that could exist in an autonomous domain which is metaphysically independent from behavior and action is brought into question by a close examination of our ordinary ways of speaking about psychological matters. To accept this is to reject the standard characterization of mind as motivated by a certain picture of our everyday psychological talk (one that pretends to derive from that everyday talk itself). That picture is what grounds the intuitions that abound about the nature of mental states, their causal efficacy, and other related claims.

The so-called commonsense functionalist characterization of inner mental states that it promotes—its conception of “mental states” as “inner causes” is not a part of commonsense, rather it is a theoretically driven picture of commonsense view of the mind. The functionalist framework is not an innocent or inevitable feature of our everyday talk about the psychological; it is not built-into that discourse, it is a presumptuous imposition upon it (Hutto, 2013). When we look (rather than “think”—that is, rather than presupposing and imposing) we find that there are no clear lines in folk talk about the mind that demarcate the boundaries of cognitive phenomena neatly (Wittgenstein, 1953). Folk talk is not designed to tell us where perceptual activity, for example, begins and ends in any precise sense. Arguably what Wittgensteinian and phenomenological investigations into our ordinary thought and talk about the mental reveal is that while the folk do conceive of cognition and perception as kinds of public activity, their spatial and temporal boundaries are messy, rough-edged and extensive (Hutto, in press). This is what justifies thinking of minds as naturally extensive—the idea that cognitive activity always already entangles embodiment, action, and world-involving resources and does not restrict itself only to what is inside the individual organism.

Does this mean the extent of cognition has no limits? Of course not. And those limits can be revealed by empirical experiment. Even if one were able to see for miles one might not be able to see for leagues. There will be empirically discoverable species-wide and individual differences with respect to the limits of cognition—limits that can and should (and are being) actively empirically explored. Rossi et al. (2014) provide an excellent example of such an exploratory project. They seek to discover how best to characterize a dog's sensitivity to, and capacity to make use of, human movements and gestures. Using a head-mounted eye-tracking system, Rossi et al. (2014) investigate dogs' response profile by discovering exactly which salient features (affordances) in the embedding environment the animal is responding to. Crucially, they found that dogs are “especially capable of utilizing human pointing gestures and of following our eye-gaze direction” (2014, p. 135). This sort of experimental work on discovering affordances promises to provide a detailed integrative account of the limits and extent of cognition amongst conspecifics and non-conspecifics (Chemero, 2009; Rossi et al., 2014). It is precisely at this sort of interdisciplinary interface that philosophy and science can and should fruitfully use to inform one another.

## Conclusion

We hope by now to have established that the notion of extensive minds is not hopelessly vague or ill-conceived. Indeed, we have argued that—even if not named—it already figures in explanations required by the sciences of the mind and our ordinary ways of understanding the mental.

As a final word, it should be clear from the discussion above, that endorsing extensive enactivism does not entail leaving neuroscience behind or out of the story. Even if the great bulk of cognitive processes are extensive and world-involving there is still every reason to understand empirically the special contributions that brains make to enabling such cognition.

### Conflict of interest statement

The authors declare that the research was conducted in the absence of any commercial or financial relationships that could be construed as a potential conflict of interest.

## Footnotes

^1^To prevent misunderstanding, it is important to clarify what “basic” cognition means on a REC account. REC promotes the possibility that there are kinds of mind that lack content, namely that non-representational minds exist. REC also allows that contentful minds exist. It assumes not only that there are a variety of types of minds (some are contentful, some not) but also that non-contentful minds are, phylogenetically and ontogenetically, basic. Non-contentful minds are the most fundamental kinds of minds. Content-involving modes of cognition are necessarily scaffolded modes of cognition. From a REC perspective contentful thought is not a feature of all forms of cognition, rather it is a special achievement. Being basic doesn't imply low-grade; there may be many quite important and sophisticated yet basic forms of non-contentful cognition.

^2^Wheeler defended this view in his closing keynote to the Varieties of Enactivism: A Conceptual Geography symposium, hosted by the AISB-50 in April 2014.

^3^Thus Wheeler (2014) claims, “one leading alternative to enactivism in 4E space, namely extended functionalism, is in the position to robustly reject [internalism about cognitive architecture] but, unlike enactivism, has no theoretical interest in rejecting [content-bearing representations]” (p. 1). Indeed he goes further and says, “Against Hutto and Myin, I shall briefly present an analysis which indicates that to the extent that extended functionalism can be adequately defended, it can be defended in a representationalist register” (p. 1).

^4^As Piccinini notes, “Many … [will assume that] computational states are individuated by their formal or syntactic properties … that computational states are individuated syntactically rather than semantically. But this is far from the case” (Piccinini, 2008, p. 208). Putting the point more softly, P observes “it has seemed difficult to give an account of syntactic properties without appealing to semantic properties (cf. Crane, 1990; Jacquette, 1991; Bontly, 1998). Nevertheless, he then adds, “I don't know how to tell whether a property is syntactic” (Piccinini, 2008, p. 209).

^5^It is not our aim here to argue that an appeal to mechanisms must fail in the debate about the extent of cognition. In fact, Craver mentions that some cognitive mechanisms make use of resources outside organismic boundaries to such an extent that “it may not be fruitful to see the skin, or the surface of the CNS, as a useful boundary” (2007, p. 141). Kaplan (2012) applies a mechanistic analysis to the extended mind theory. Yet for all this, a mechanism is—as Bechtel (2008) mentions—a structure that performs a specific role or function in virtue of its parts, their operations, and their joint organization. In the cognitive domain, Bechtel states, what is distinctive of cognitive mechanisms—as opposed to non-cognitive mechanisms—is that the operations of component parts involve information processing, which suggests that information is what is operated on. However, if individuating cognitive mechanisms from non-cognitive ones turns on a theory of content, and if basic cognition is non-representational and therefore non-contentful, then cognitive mechanisms cannot be individuated solely by appealing to certain kinds of contentful operations; this follows independently of the debate about where to draw the boundaries of cognition.

^6^There is evidence of the inherent circularity in this sort of approach in Rupert's official formulation: “If there is any theoretically interesting divide between what is distinctively cognitive and what merely causally contributes to intelligent behavior, it is to be found in the persisting, integrated nature of cognitive architectures” (Rupert, 2010, p. 344, emphasis added).

^7^Evidence that such empirical assumptions are being made is easy to find: “We think orthodox cognitive psychology places its bets on the individuation of cognitive processes in terms of mechanisms.” (Adams and Aizawa, 2008, p. 125). “We think that the available empirical evidence provides good reason to think that the chances of finding interesting cognitive regularities covering brains and tools is low. Bear in mind that we side with what is by all accounts scientific orthodoxy.” (Adams and Aizawa, 2010, p. 74, emphasis added).

^8^Chemero (2009) reports that, “the main results of the literature survey were as follows. Of these 116 articles, 52 (approximately 44%) gave little or no information concerning the specific objects that were given for exploration, and 64 (approximately 56%) provide detailed descriptions of the objects. Of the 64 articles that included descriptions of the objects used in the experiments, 32 (approximately 28% of the total) used sets of objects with non-equivalent affordances for the species of animal under study” (Chemero, 2009, p. 173).

^9^Clark (2011) observes that one of Wheeler's (2011) reasons for steering clear of commonsense functionalism is that: “Direct appeal to the folk intuitions, Wheeler claims, will either yield the wrong results (because the folk are basically internalists at heart), or will be of little value anyway, in debates concerning vehicles or realizers. Instead of appealing to the unregimented folk intuitions, then, Wheeler suggests we should see ‘a scientifically informed, theory-loaded, locationally uncommitted account of the cognitive” (p. 451).

^10^We agree with Clark (2011) that: “There is no need for a mark of the cognitive … because we already have an implicit (though probably unformalizable in words) grip on the kinds of coarse-grained behavior patterns that we take to be indicative of key mental states, such as the holding of a standing (dispositional) belief” (Clark, 2011, p. 451; emphasis original).
